# Optimization of miRNA-seq data preprocessing

**DOI:** 10.1093/bib/bbv019

**Published:** 2015-04-17

**Authors:** Shirley Tam, Ming-Sound Tsao, John D. McPherson

**Keywords:** miRNA sequencing, data preprocessing, small RNA sequence alignment, miRNA-seq normalization

## Abstract

The past two decades of microRNA (miRNA) research has solidified the role of these small non-coding RNAs as key regulators of many biological processes and promising biomarkers for disease. The concurrent development in high-throughput profiling technology has further advanced our understanding of the impact of their dysregulation on a global scale. Currently, next-generation sequencing is the platform of choice for the discovery and quantification of miRNAs. Despite this, there is no clear consensus on how the data should be preprocessed before conducting downstream analyses. Often overlooked, data preprocessing is an essential step in data analysis: the presence of unreliable features and noise can affect the conclusions drawn from downstream analyses. Using a spike-in dilution study, we evaluated the effects of several general-purpose aligners (BWA, Bowtie, Bowtie 2 and Novoalign), and normalization methods (counts-per-million, total count scaling, upper quartile scaling, Trimmed Mean of M, DESeq, linear regression, cyclic loess and quantile) with respect to the final miRNA count data distribution, variance, bias and accuracy of differential expression analysis. We make practical recommendations on the optimal preprocessing methods for the extraction and interpretation of miRNA count data from small RNA-sequencing experiments.

## Background

microRNAs (miRNAs) are small non-coding RNAs (∼18–22 nucleotides in length) that negatively regulate gene expression by binding to the 3′ UTRs of target genes. Depending on the degree of sequence complementarity, this interaction can mediate either translational repression or mRNA degradation. More than 30% of human protein-coding genes are predicted to be conserved targets of miRNAs [1]; a single miRNA may target many hundreds of genes, potentially disrupting entire gene networks. This dysregulation may, in turn, be reflected in the expression patterns of one or just a few miRNAs. By comparing miRNA profiles between different states, such as ‘experimental’ versus ‘control’, we can identify patterns or specific miRNAs implicated in different biological processes or disease pathogenesis.

With the decreasing cost and high multiplexing capability of next-generation sequencing (NGS), this technology is increasingly being used for the comprehensive profiling of miRNA abundance. Although several platforms and protocols exist, including the Illumina HiSeq systems, Life Technologies SOLiD™ sequencing and Roche 454 sequencing, the analysis of the resulting data follows a general scheme: (1) short reads are filtered for low-quality sequences and adapters, (2) the remaining sequences are mapped to a reference (genome, miRBase, non-coding RNAs, Refseq and so on), (3) the abundance for each biological entity of interest is determined (to give an expression measure of specific targets), (4) the resulting count data are normalized and (5) downstream analyses are conducted to probe biologically relevant questions. Despite this, no standard method or clear consensus exists on how the data should be preprocessed and the consequences of a chosen method on subsequent analyses.

Several software packages have been developed over the past few years for the preprocessing and mapping of miRNA-seq data [2–13]. These tools differ in the methods used for adapter trimming or clipping, types and thresholds used for filtering and alignment algorithms. Of these steps, the choice of the alignment algorithm will have the greatest impact on the recovery of accurate miRNA abundance profiles of the sequenced samples. Although different aligners have been optimized with respect to various considerations, they may not all be suitable for miRNA-seq data alignment. For example, SOAP [14] was specifically designed for detecting single-nucleotide polymorphism, whereas mrsFAST [15] was developed primarily for the detection of structural variants [16]. Testing different software for miRNA-seq alignment can help select an optimal aligner for this type of data.

Following alignment, the recovered miRNA counts need to be normalized to remove variations in the data that are of non-biological origins and can affect the measured abundance levels. Even in replicate experiments, some variations will be observed, stemming from the experimental procedure—sample handling, library preparation and sequencing. An effective normalization technique should minimize technical and experimental bias without introducing noise. The differences that remain should be truly biological effects. Several normalization methods for miRNA-seq data have been proposed, including linear scaling, nonlinear scaling and quantile normalization. However, no standard method is currently used; read counts from each experiment are usually simply adjusted for differences in sequencing depth to counts-per-million (cpm), despite studies showing that this is insufficient to account for the technical differences across samples [17, 18]. These two studies, comparing the effect of different normalization methods, made conflicting conclusions: Garmire and Subramaniam supported the use of locally weighted linear regression (Lowess) and quantile normalization, while discouraging against trimmed mean of M (TMM)—these results were validated using polymerase chain reaction (qPCR); and Dillies *et al*. suggested the opposite, advising against quantile normalization but advocating for the TMM method—data simulations were used to confirm these findings. While different normalization techniques will likely affect the accurate quantification of miRNA abundance, the downstream consequences of using different aligners to map the raw sequencing reads to a given reference is unknown: the former study used data aligned using SHRiMP [19] and Bowtie [20], whereas the latter used Novoalign [21]. Data normalization will not rescue discrepancies caused by the initial alignment.

To assess the relative merits of different preprocessing methods in terms of variance, bias and sensitivity and specificity for the detection of differential expression, data for which true values are known are required [22]. Here, we generated a spike-in data set, whereby known amounts of oligonucleotides were added to a common biological reference background. Using this data set to simulate data characteristics observed in actual experiments, we assessed the impact of alignment and normalization on the final processed data and downstream analyses. Our findings were then validated on a previously published data set, which compared miRNA profiles between cell lines and their corresponding xenografts.

## Methods

### Data sets

Two data sets were used for this study: a spike-in experiment and a miRNA-seq profiling data set comparing cell lines and xenografts. The spike-in data set was created using a 12 × 12 cyclic Latin Square design. Twelve miRNAs from the *Arabidopsis thaliana* genome that are not present in the human genome were selected as spike-in sequences. RNA oligonucleotides were synthesized with phosphorylated 5′ ends (Integrated DNA Technologies, see Supplementary Table S1 for sequence information), and added at 12 different concentrations (0, 0.1, 0.2, 0.8, 1.6, 6.4, 12.8, 51.2, 102.4, 409.6, 819.2, 3276.8 amol) to 1 μg Universal Human Reference RNA (Agilent Technologies), with each concentration appearing once in each row and column of the design matrix. The samples were subjected to all experimental steps, including small RNA purification. The second data set, consisting of miRNA-seq data from matched cell lines and xenografts, has been previously published [23]; this data set is publicly available in the Gene Expression Omnibus (GEO) repository under the accession GSE51508. This data set was chosen because qPCR data were also generated, which allowed for the assessment of sensitivity and specificity.

### Overview of the Illumina system

cDNA libraries for sequencing were constructed as per Illumina’s TruSeq Small RNA protocol (Illumina). In brief, 3′ and 5′ adapters were sequentially ligated to the ends of RNA < 200 nt long, fractionated from 1 μg of total RNA (PureLink miRNA Isolation kit, Life Technologies) and reverse transcribed to generate cDNA. The cDNA was amplified (11 cycles of PCR) using a common primer complementary to the 3′ adapter, and a 5′ primer containing 1 of 48 index sequences. Samples were size-selected on a 6% polyacrylamide gel, purified, quantified and pooled for multiplexed sequencing. The resulting pooled libraries were hybridized to oligonucleotide-coated single-read flow cells for cluster generation on-instrument and subsequent sequencing on an Illumina HiSeq 2500 instrument. Fifty sequencing cycles were performed.

### Sequence alignment

Short read sequences were output in FASTQ format with corresponding base quality scores. The raw data were initially filtered for reads containing ambiguous base calls, which did not meet the Illumina chastity filter based on quality measures. Quality control of the remaining sequences from each sequenced library was investigated using FastQC (v0.11.2) [24] to check for homopolymers, adapters and distribution of base quality. The reads were then filtered for low-quality reads, contaminating 5′ adapters, homopolymers and trimmed for 3′ adapters. The preprocessed reads were aligned in a sequential manner: first to ribosomal RNA/repeats, miRBase v20, RefSeq and finally to a genomic reference (hg19). The spike-in sequences were included in the miRBase v20 reference before indexing. Several general-purpose aligners were evaluated, including BWA [25] and Bowtie [20], the two most highly cited aligners, Bowtie 2 [26], and Novoalign [21], a propriety software from Novocraft, which is becoming quite popular owing to its high accuracy claim. Sequence alignment was performed with the following parameters for each aligner, respectively:

1. BWA 0.7.4: bwa aln -n 1 -o 0 -e 0 -k 1 -t 4
2. BWA 0.7.4 (0 mismatch in seed): bwa aln -n 1 -o 0 -e 0 -l 8 -k 0 -t 4
3. Bowtie 0.12.9: bowtie -n 1 -l 8 -a --best --strata --phred33-quals
4. Bowtie 0.12.9 (0 mismatch in seed): bowtie -n 0 -l 8 -a --best --strata --phred33-quals
5. Bowtie2 2.1.0: bowtie2 --local -p 8 -q --phred33 -D 20 -R 3 -N 0 -L 8 -i S,1,0.50
6. Novoalign 3.00.05: novoalign -a TGGAATTCTCGGGTGCCAAGG -l 15 -t 30 -r A

Only reads uniquely aligned to miRNAs were retained and counted. The miRNAs were filtered for targets with a minimum of 5 counts in at least 25% of the samples. The raw and processed data has been deposited in the Gene Expression Omnibus repository, under the accession GSE67074.

### Normalization

Several normalization methods were evaluated, including (1) cpm, (2) total count scaling, (3) upper quartile scaling (UQ), (4) TMM, (5) DESeq, (6) linear regression, (7) cyclic loess normalization and (8) quantile-based normalization. Each of these methods is described briefly.

(1)* Count-per-million—*the simplest form of normalization, whereby each library is adjusted for differences in sequencing depth. The counts can then be adjusted to reads per million to facilitate comparison between samples.

(2)* Total count scaling—*After scaling each sample to its library size, they can be rescaled to a common value across all samples. The baseline reference can be chosen to be the sample with the median library size. If *s_baseline_* is the size of the reference library, and *s_i_* is the sum of all reads of the any given library, then the normalization factor is as follows:

(1) $$d_{i}=\frac{s_{baseline}}{s_{i}}$$

and the counts for the normalized samples would be

(2) $$x_{i}^{\prime }=d_{i}x_{i}$$

where *x_i_* is the raw count for a specific target.

(3)* Upper-quartile scaling*—In RNA-seq experiments, the predominance of zero and low-gene counts has led to the suggestion of a modified quantile-normalization method: the upper quartile of expressed miRNAs is used instead as a linear scaling factor [27]. This method has been shown to yield better concordance with qPCR results than linear total counts scaling for RNA-seq data [27]. It is expected that in miRNA-seq experiments, the 75^th^ percentile of the data will also be found at only 1 or 2 copies/library.

(4)* Trimmed mean of M*—Normalization by total count scaling makes intuitive sense because it gives us the proportion of counts for a specific target across all samples. If a miRNA is present in the same proportion across all samples, it will be deemed as non-differentially expressed. However, this method does not take into consideration the potentially different RNA composition across the samples. TMM, proposed by Robinson *et al*. for RNA-seq data normalization, calculates a linear scaling factor, *d_i_*, for sample *i*, based on a weighted mean after trimming the data by log fold-changes (*M*) relative to a reference sample and by absolute intensity (*A*) [28]. TMM normalization takes into account the composition of the RNA population being sampled, which is neglected in total count scaling. This method is implemented in the R Bioconductor package edgeR, with default trimming of M-value by 30% and A-values by 5% [29].

(5)* DESeq*—To perform differential expression analysis using count data, Anders and Huber proposed modeling the data with the negative binomial distribution, and incorporating data-driven prior distributions to estimate the dispersion and fold changes [30]. As a data preprocessing step, the authors introduced the size factor—a scaling factor—to bring the count values across all the samples to a common scale. The size factor for a given library is defined as the median of the ratios of observed counts to the geometric mean of each corresponding target over all samples. This method is implemented in the R Bioconductor package DESeq.

(6)* Linear regression—*This normalization technique assumes that the systematic bias is linearly dependent on the count abundance. In microarray data, the log2 ratios, *M*, between two samples have been observed to have systematic dependence on the intensity values, *A*. This can be visualized by plotting the M values for each element as a function A (MA plot), where

(3) $$M_{i}=\log_{2}\left(\frac{x_{i,j=1}}{x_{i,j=2}}\right)$$

and

(4) $$A_{i}=\frac{1}{2}\log_{2}\left(x_{i,j=1}x_{i,j=2}\right)$$

with *x_i.j_* as the abundance for a given miRNA, *i*, in sample *j* [31]. A linear least squares regression model is applied in the form of [32]

(5) $$M^{\prime }=\beta_{0}+\beta_{1}A$$

The normalized ratio is calculated from the regression equation [33],

(6) $$M_{i}^{\prime }=M_{i}-M_{i}^{*}$$

where $M_{i}^{*}$ is the predicted ratio from the least squares estimation. The normalized *x_i.j=__1_ and xi_.j=__2_* values are given by [33]

(7) $$x_{i,j=1}^{\prime }=2^{a+\frac{1}{2}m_{i}^{\prime }}\text{ and }x_{i,j=2}^{\prime }=2^{a-\frac{1}{2}m_{i}^{\prime }}\text{.}$$

The samples were normalized to a baseline reference, which was defined as the median count of each element across the profiled samples.

(7)* Nonlinear regression*—Although linear normalization is simple and robust, the linearity assumption does not always hold, especially at extreme values. Lowess analysis was initially proposed for the removal of intensity-dependent effects of the log2 ratios in two-color microarray experiments [34]. A weight function is determined, which puts less emphasis on the contribution from elements that are far from each point on a MA plot [35]. The regression model for Lowess normalization can be considered to be in the form of [32]

(8) $$M^{\prime }=M-c(A)$$

where c(A) is given by the robust scatter plot smoother Lowess. The normalized M ratios are determined in the same manner as in linear regression normalization. For the normalization of miRNA-seq data, a cyclic loess approach was used, initially described by Boldstad *et al.* for single channel microarrays [33].

(8)* Quantile-based normalization—*Initially proposed for the normalization of microarray data, quantile normalization forces the distribution of read counts in all samples across an experiment to be equivalent [33]. This non-scaling approach assumes that most targets are not differentially expressed and that the true expression distribution is similar across all samples.

### Data analysis

Data analyses and graphical representations were performed and generated in the R statistical environment (v3.1.2). Normalization methods were implemented either in R or Bioconductor (3.0) libraries. Cyclic loess and quantile normalization were performed using the normalizeBetweenArrays()function in the limma package (v3.22.4) [36], whereas TMM and UQ normalizations were performed using calcNormFactors() in the edgeR (v3.8.5) package [29]. The function estimateSizeFactors() in the DESeq (v1.18.0) package was used to normalize the count data using size factors [30]. For differential expression analysis of the qPCR data in the cell lines–xenografts comparison study, linear models were fit to each miRNA, and an empirical Bayes approach was applied to moderate the variance [36]. The same linear modeling was applied to the sequencing data, but the mean-variance trend was first estimated from the data and incorporated into a precision weight for each individual observation [37]. A paired sample design was used for all analyses, matching each cell line with its respective xenograft model. The *P*-values were adjusted for multiple testing using the Benjamini and Hochberg approach. This analysis was performed using the limma package (v3.22.4).

## Results

### Comparison of alignment tools

**Figure 1.**
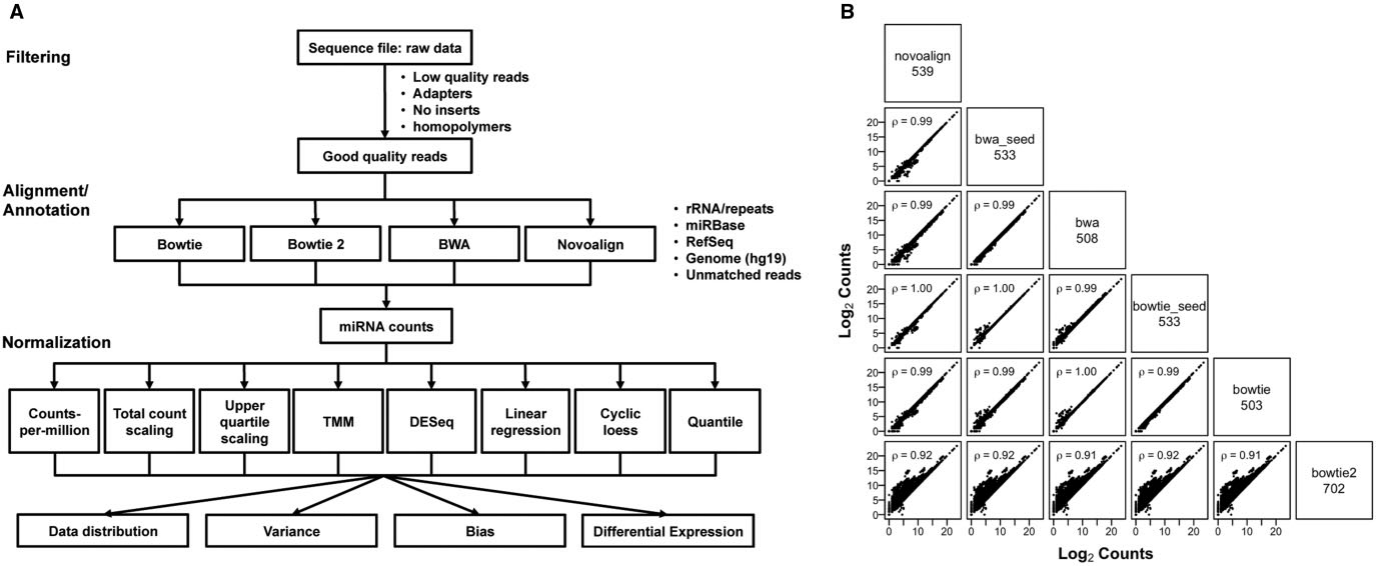
(**A**) Outline of the experimental design and workflow for evaluating different combinations of alignment tools and normalization procedures. (**B**) Comparison of alignment tools. miRNA counts recovered from the different aligners were averaged across the samples and plotted for each pairwise comparison. Only miRNAs called present by all the aligners were considered. The aligners being compared are shown on the diagonal, along with the distinct number of miRNAs identified after filtering for species with low counts. Spearman’s correlation coefficients are indicated in the upper left corner for each comparison.

Table 1 lists the attributes of software packages and methods that have been developed over the past few years for the preprocessing and mapping of miRNA-seq data. Aligners used in the more recently developed software were compared, including BWA, Bowtie and Novalign (see Figure 1A for experimental design). The alignment parameters were set to be as similar as possible across the different tools when permitting. For example, BWA and Bowtie were run with two different settings: (1) allowing one mismatch across the entire read including the seed region (bwa_seed and bowtie_seed) and (2) allowing one mismatch in the non-seed region only. The distribution of reads mapping to the different annotated references is quite similar (see Supplementary Figure S1). Despite this, the resulting Bowtie output had a number of miRNAs that were estimated to have higher abundance levels than the count estimates from BWA and Novoalign, which was run with the recommended parameter settings for miRNA analysis (Figure 1B). This effect was even more pronounced in the Bowtie 2 output. Overall, the miRNA abundance profiles were highly similar, with Spearman’s ρ ≥ 0.91. Following alignment, the recovered miRNA abundance counts were normalized using the different methods described in the Methods section and used for all subsequent analyses.

**Table 1.** Software packages and methods for miRNA-sequencing data preprocessing and alignment

	Preprocessing			Annotation	
	Adapters	Filtering	Output	Algorithm	Reference
Morin et al., (2008)		Trim all reads to 30 nt	Count file	MegaBlast	Genome Annotate genomic positions to miRNA, tRNA, rRNA, scaRNA…
SeqBuster (2009)	• Modification of Needleman-Wunsch -3′ adapter removal			MegaBlast	User-supplied databases
miRExpress (2009)	• 3′ adapter trimming	Reads with 3′ adapters in the middle or beginning	Count file	Smith-Waterman	miRBase
E-miR (2010)	• Regular expression matching - 3′ adapter removal	Reads <15 nt Trim reads >32 nt	Count file	Eland	Genome Annotate genomic positions to non-coding RNA transcripts
mirTools (2010)	• Custom Script - 5′/3′ trimming	PolyA Quality	Collapsed fasta	SOAP	Genome miRBase, repeats, coding genes…
DSAP (2010)	• Supermatcher - 5′/3′ clipping	Homopolymers Reads <16 nt Read with no 3′ adapter	Collapsed tags	BLAST	ncRNAs: rRNA, tRNAs, snRNAs, snoRNAs… miRNA
miRNAkey (2010)	• 3′ adapter clipping	Read length post-clipping		BWA	miRBase
Flicker (2011)	• 3′ adapter trimming		Fasta	Eland	Contaminants (rRNA, primers…) Genome miRNA miRNA precursors
miRanalyzer (2009, 2011)	• Optional adapter removal	‘N’ containing reads Reads <17 nt Trim reads >26 nt	Count file Multi-fasta	Bowtie	miRBase: mature, precursor RefSeq, Rfam Genome
miRDeep2 (2011)	• Custom script - Clip 3′ adapter	Reads <18 nt	Collapsed reads	Bowtie	Genome Map miRNA and reads to precursors; find intersection
shortran (2012)	• FASTX trimming	Abundance Inter-library variation Sequence (Bowtie)	Multi-fasta	Bowtie	Genome User-supplied sequence files
Farazi *et al*., (2012)	• Custom script -3′ adapter trimming	Reads <16 nt and >25 nt Mono-, di-, tri-nucleotide repeats Reaction by-products (Needleman–Wunsch algorithm)	Collapsed fasta	BWA	Genome Map unique sequences to rRNA, tRNA, sn/snoRNA, repeats, miRNA…

### Qualitative assessment of normalized data

**Figure 2.**
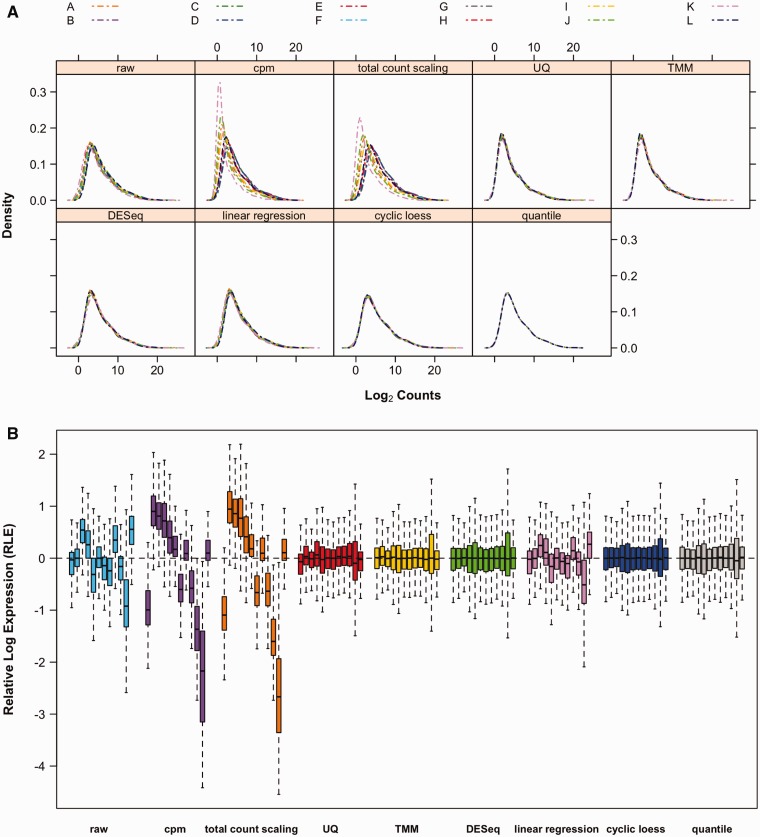
Comparison of data distribution. (**A**) Density plots of log count distribution. Distributions of the samples before and after normalization are shown in different panels for each normalization method. Comparing between samples, none appears to have abnormal distributions. (**B**) Boxplots of RLE. The RLE distributions of comparable samples should be centered at zero and similar to each other. Boxplots of the raw data clearly indicate the need for normalization. Values beyond the 1.5 interquartile range (IQR) are not shown. The samples are grouped according to the normalization method, with the order of the samples consistent across each group. A colour version of this figure is available at BIB online: http://bib.oxfordjournals.org.

As an illustration of the different normalization methods, the absolute distribution of the miRNA count data following alignment and normalization can be visualized using density distribution curves (Figure 2A). To avoid problems associated with zero values, the data were log2 transformed after the addition of +1 to all counts. From the density curves of the raw counts, it is evident that there are some inconsistencies between the distribution profiles of the samples. Adjusting the data by cpm or total count scaling introduces more variability to the data, whereas all other methods resulted in more similar distribution across all samples. The relative log expression (RLE), defined as the difference between the log of a read count and the log of the median count across the samples, should be centered at zero and have comparable distributions across similar samples. The boxplot of the unnormalized RLE shows large distributional differences across the samples (Figure 2B); these differences are amplified in data normalized by cpm and total count scaling. The distributions are more similar and centered at zero when the data are normalized using UQ, TMM, DESeq, cyclic loess and quantile normalizations. Figure 2 shows the difference in normalization of the data aligned using BWA with one mismatch allowed across the entire read. The same assessment performed on the data generated from the other five alignment outputs showed similar results (data not shown), most likely owing to the comparable percentage of reads mapping to miRNAs across the different aligners (Supplementary Figure S1).

### Variance comparison

**Figure 3.**
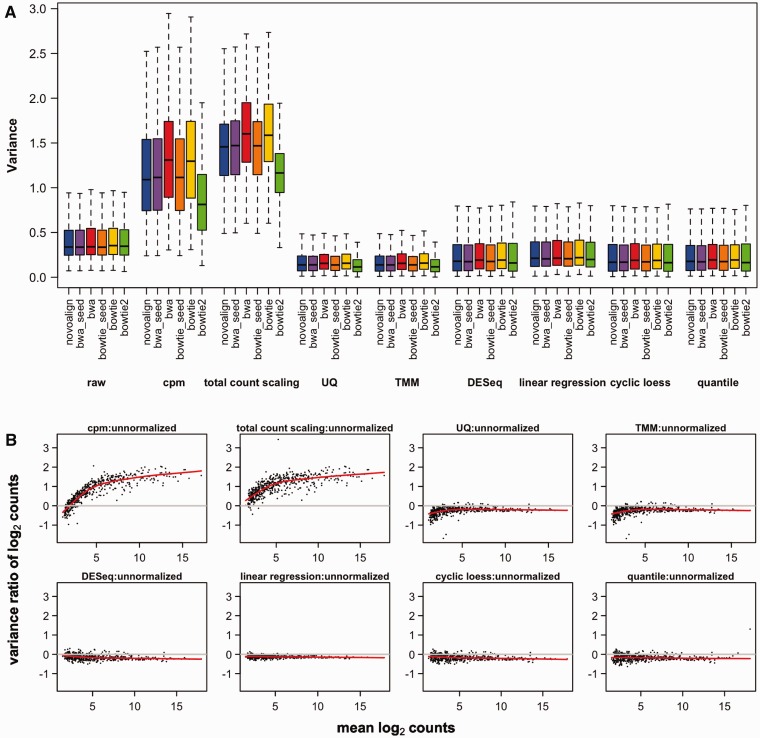
Variance comparison. (**A**) Boxplots of the variance distribution. The variance of the log2 counts of all non-spike-in miRNAs was computed across the samples, and visualized using boxplots. The data are grouped according to the normalization method. Values beyond the 1.5 interquartile range (IQR) are not shown. A clear increase in variance is observed in the data normalized by cpm or total count scaling, while a decrease is seen in UQ or TMM normalized data. (**B**) Mean-variance dependency. The relationship between variance and abundance level is visualized by plotting the ratio of the variance between the normalized and unnormalized data versus the average counts. Data points below the y = 0 line have decreased variance compared with the raw data. The Lowess smoother line reveals the presence of any trends in the data. A colour version of this figure is available at BIB online: http://bib.oxfordjournals.org.

As the samples were prepared using the same background reference, most of the detected species should have similar abundance levels, except for the 12 spike-in sequences. Adjusting the data by cpm or total count scaling introduces more variability, whereas UQ and TMM decreased the variance across all miRNAs compared with the raw data (Figure 3A). The percentage of miRNAs with decreased variance following normalization by each of the methods is shown in Table 2.

**Table 2.** Change in variance and assessment of bias post-normalization

	% with lower variance						Median slope (β_1_)*						Median *R*^2^*					
	Novoalign	BWA seed	BWA	Bowtie seed	Bowtie	Bowtie2	novoalign	BWA seed	BWA	Bowtie seed	Bowtie	Bowtie2	novoalign	BWA seed	BWA	Bowtie seed	Bowtie	Bowtie2
Raw		–	–	–	–	–	0.96	0.96	0.98	0.96	0.97	0.96	0.96	0.96	0.98	0.96	0.97	0.96
Cpm	15.4	15.0	10.5	15.5	10.4	24.8	0.87	0.87	0.88	0.87	0.87	0.86	0.87	0.87	0.88	0.87	0.87	0.86
total count scaling	1.1	1.0	0.6	1.0	0.6	1.2	0.92	0.92	0.91	0.92	0.91	0.92	0.92	0.92	0.91	0.92	0.91	0.92
upper quartile	96.4	96.5	96.6	96.5	96.7	97.2	0.97	0.97	0.97	0.97	0.97	0.97	0.97	0.97	0.97	0.97	0.97	0.97
TMM	95.4	95.4	95.6	95.4	95.7	97.2	0.97	0.97	0.97	0.97	0.97	0.97	0.97	0.97	0.97	0.97	0.97	0.97
DESeq	86.3	85.4	88.9	85.0	88.6	88.7	0.98	0.98	0.99	0.98	0.99	0.98	0.98	0.98	0.99	0.98	0.99	0.98
linear regression	95.1	95.6	95.8	95.4	95.9	94.9	0.97	0.97	0.97	0.97	0.97	0.98	0.97	0.97	0.97	0.97	0.97	0.98
cyclic loess	88.0	87.7	89.3	87.9	89.2	88.7	0.98	0.98	1.00	0.98	0.99	0.98	0.98	0.98	1.00	0.98	0.99	0.98
Quantile	88.8	88.1	89.5	87.5	89.2	88.6	0.95	0.96	0.96	0.96	0.96	0.97	0.95	0.96	0.96	0.96	0.96	0.97

*The ath-miR405a dilution series was excluded from the analysis of bias.

To identify any dependency on abundance levels, the mean and variance of each miRNA was computed across the samples, and the log ratio of the variance of normalized versus unnormalized data was plotted against the mean abundance levels (Figure 3B). From the Lowess smoother, the variance introduced by cpm and total count scaling increases slightly with increasing counts, whereas no such trend is present in the data normalized using all other methods. UQ and TMM normalizations appear to have a slight edge over the other methods at the lower range.

### Assessment of bias

An effective normalization method should decrease the variance without increasing bias. The following linear model was fit to each spike-in dilution series to assess the bias in the preprocessed data:

(9) $$\log_{2}C=\beta_{0}+\beta_{1}\log_{2}a+\varepsilon$$

where *C* is the count value and *a* is the amount, in attomole, of oligonucleotides added. The sample with spike-in concentration of 0.0 amol was excluded from the model fit. The closer the slope (β_1_) is to 1, the more the data are representative of the true values. The *R*^2^ value was determined to assess the model fit.

**Figure 4.**
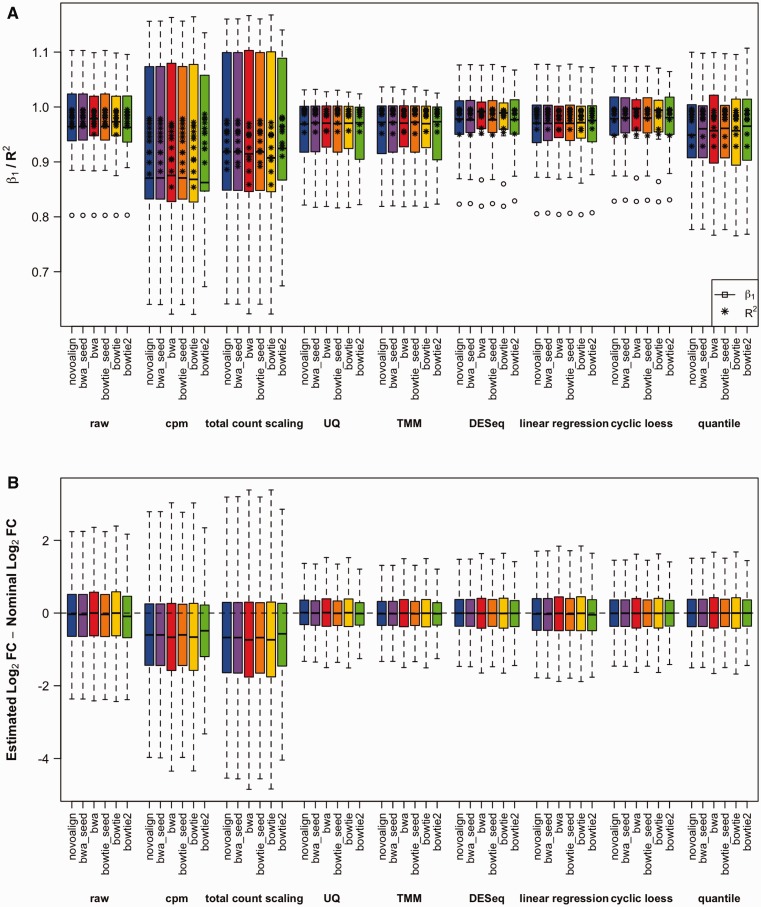
Bias assessment. (**A**) Assessment of bias. Linear regression was performed on the spike-in dilution series, and the resulting slopes (β_1_) were visualized using boxplots, with the asterisk symbol (*) representing the *R*^2^ values. The data are grouped according to the normalization method. (**B**) Comparison of miRNA-seq fold-changes and nominal fold-changes. The difference between experimentally determined fold-changes and nominal fold-changes was determined for all miRNAs. Boxplot show the distribution of the deviation from the expected fold-changes. The data are grouped according to the normalization method. A colour version of this figure is available at BIB online: http://bib.oxfordjournals.org.

For the data normalized by UQ and TMM, β_1_ for the spike-in dilution series are closer to 1; however, increase in bias is observed in the data adjusted by cpm, total count scaling and quantile normalization compared with the raw data (Table 2). The distributions of the β_1_ and *R*^2^ values are shown in Figure 4A. A problematic dilution series (ath-miR405a) was identified with low *R*^2^ and β_1_ values, which is most likely attributed to pipetting errors. This sequence was removed from this analysis and all subsequent analyses. For the remaining spike-in sequences, some species have slopes >1, whereas others have slopes <1. Although the most obvious explanation is the presence of experimental errors, this effect can also be a result of the background noise, such as sequencing error, random sampling or misalignment. For example, as sequencing reads are a random sample of the population of transcripts, the lower abundance species may be less likely detected; this will cause *β*_1_ > 1. On the other hand, *β*_1_ < 1 may be a result of the alignment of reads with a single base mismatch; imperfect alignments will have a more dramatic effect on low abundance species.

The absolute bias was calculated as the difference between the estimated fold-changes and the nominal fold-changes. The background miRNAs are expected to remain consistent across all the samples, while changes in abundance of the spike-in sequences between samples are known. Ideally, the absolute bias should be centered at zero (Figure 4B). While normalization by cpm and total count scaling resulted in increased bias compared with the raw data, UQ, TMM, DESeq and cyclic loess reduced the bias closer to zero.

### Differential expression analysis

**Figure 5:**
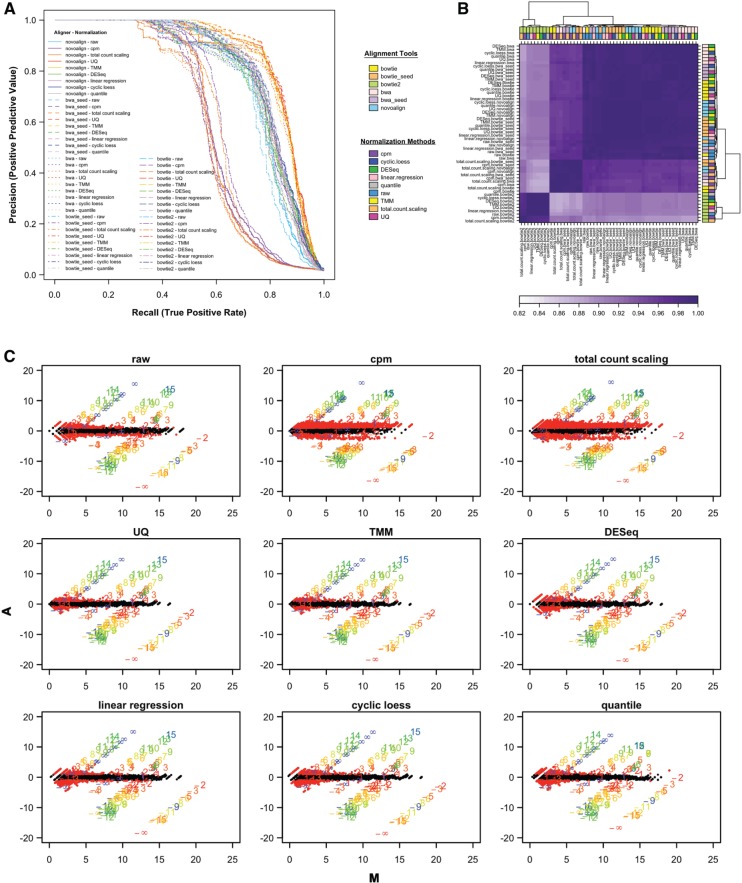
Accuracy in predicting differential expression. (**A**) PR curves. The effect of normalization on the accuracy of differential expression analysis is visualized using PR curves. miRNAs with a fold-change ≥2 were considered to be differentially expressed. The spike-in sequences and the background reference were used as the true-positive results and true-negative results, respectively. Colors represent different normalization methods, while lines represent aligners. Please see online version for coloured image. (**B**) Comparison of fold-change estimates. Spearman’s correlation coefficients between fold-change estimates obtained from the differently processed data were calculated and subjected to unsupervised hierarchical clustering using Euclidean distance as the distance metric and complete linkage. (**C**) M versus A plots. MA plots were generated by comparing all samples to one chosen reference sample. The black points represent log2 fold-changes from the background, while the red points represent the non-spike-in miRNAs that have a fold-change ≥2. The colored numbers represent the spike-in sequences, with the precise number representing the expected fold-change. A colour version of this figure is available at BIB online: http://bib.oxfordjournals.org.

Data preprocessing procedures can also be judged by the improved sensitivity and specificity for the detection of differential expression. As sequences were spiked into a common background reference, successful differential expression analysis should only identify the spike-in sequences as differentially expressed. Following normalization, log2 ratios for all pairwise comparisons were computed, and precision (positive predictive value) and recall (true positive rate) were determined for a range of thresholds for all data sets. As the smallest difference in the amount of spike-in sequences added across the samples is 2 folds, miRNAs with a fold-change ≥2 were considered to be differentially expressed. Precision-recall (PR) curves were used to assess the performance of the different preprocessing methods in calling miRNAs differentially expressed. When the number of positive and negative examples is highly imbalanced, PR curves are better performance estimators than receiver-operating characteristic curves. An ideal PR curve will have a precision value of 1 for all values of recall. Data normalized by cpm or total count scaling performed unequivocally worse than the raw data, which were normalized by library concentration before sequencing. Data normalized by UQ or TMM clearly dominated the PR curves, with BWA-aligned reads (with 1 mismatch in the seed regions) showing a slight advantage (Figure 5A). Comparison of the estimated fold-changes from the output of different aligners and normalization procedures reveals that UQ, TMM, DESeq, quantile and cyclic loess normalized data have the most similar fold-change estimates (Figure 5B). The data aligned using Bowtie 2, regardless of the normalization methods, were different from the remaining combinations of aligners and normalization techniques used.

The increased sensitivity and specificity of these particular methods can be assessed using M versus A plots; one sample was selected as a reference, and the mean and average for each miRNA was computed for all comparison of this sample to all other samples (Figure 5C). Data normalized by UQ and TMM have smaller variances, especially for low abundance species. This is in contrast to cpm- and total count scaling-adjusted data, which have increased variance compared with the raw data across all abundance levels. Fold-change compression is also observed in the data normalized by these two methods, and by quantile normalization.

### Data normalization and sequencing depth

For data with similar library sizes, the raw and normalized data have comparable distributions, regardless of the normalization technique used (Supplementary Figure S2a). However, when large differences in read depth exist, the efficiency of the different normalization techniques at stabilizing the read distribution across a data set differs (Supplementary Figure S2b). We explore this concept using the cell lines–xenografts comparison study because of the more variable count distribution and higher coverage compared with the spike-in experiment (∼116 million total reads compared with ∼44 million reads across all samples). The read depths in this data set ranged from over 2 to 16 million reads per sample, whereas the library sizes in the spike-in experiment ranged from over 1 to 11 million reads per sample. To assess the impact of read depth on data normalization, subsampling was performed to simulate data with similar read counts across all the samples (∼2.2 million reads/samples). The effect of alignment and normalization was assessed on this subsampled and full data set using similar metrics as was performed for the spike-in experiment.

In accordance with the results observed in the spike-in data set, adjusting the data by UQ and TMM normalization resulted in more similar count distributions and RLE values centered at zero, without forcing the distribution to be exactly the same across the data set, as in quantile normalization (Supplementary Figures S3a and S3b). Unsurprisingly, the subsampled data showed comparable RLE distributions regardless of the normalization method used (Supplementary Figure S3c). Decrease in variance was only observed in data adjusted by cpm, UQ and TMM in both the full and subsampled data set (Supplementary Figure S4). To assess the presence of bias introduced by data normalization, log2 ratios for each matched cell line and xenograft pair were calculated for 56 miRNAs that were assayed and detected by qPCR. The absolute bias was defined as the deviation of these ratios from those determined by qPCR (Supplementary Figure 5). The unnormalized raw counts showed the largest amount of bias compared with the normalized data. The unnormalized subsampled data, on the other hand, had a bias distribution centered at zero. While cpm, UQ and quantile normalization successfully reduced the bias in the full data set, most normalization methods had little effect in reducing bias in the subsampled data.

Finally, the sensitivity, specificity and accuracy for the detection of differential expression can be assessed relative to qPCR, the current gold standard for validating expression profiling results. Regardless of the normalization method, data aligned using novalign resulted in lower accuracy because of the lower sensitivity of detection (Supplementary Table S2). Normalization by cpm, UQ and TMM resulted in similar sensitivity, specificity and accuracy for differential expression analysis, whereas quantile normalization showed reduced specificity. The subsampled data did not perform well in this analysis, despite the superior results observed in the assessment of bias and variance.

## Discussion

Despite the increasing use of NGS for the expression profiling of miRNAs, a standard data preprocessing pipeline for miRNA-seq data has yet to be implemented. A large number of tools and software packages are available, but the method chosen for a particular study is often left to the discretion of the researcher. To maintain consistency and reproducibility across different studies, the implementation of a standardized procedure would be critical.

The past two decades of experience with microarrays has shown that normalization is a crucial step before data analysis—subsequent analyses, such as the detection of differential expression, are highly dependent on the chosen normalization method [38]. However, it is important to note that complex methods do not necessarily perform better than simple ones—they can add noise and bias if incorrect assumptions are made. An optimal method would reduce variance without increasing bias [33]. Furthermore, a survey of published tools for the analysis of small RNA-seq data sets reveals that different aligners are embedded in these toolkits (see Table 1). The effect on the accuracy of the recovered miRNAs using different aligners is not known. To assess the effects of different preprocessing methods on the estimates of miRNA abundance in small RNA-seq data sets, we first generated a spike-in data set, whereby known amounts of 12 oligonucleotides were added to a common biological background reference. The raw sequence reads were first filtered for low-quality reads and adapters, and then aligned using BWA, Bowtie, Bowtie 2 and Novoalign. The recovered miRNA counts were normalized using cpm, total count scaling, UQ, TMM, DESeq, linear regression, quantile and cyclic loess. Normalization to a single housekeeping miRNA was not considered, as it is not known a priori which targets have stable expression levels across the samples. Furthermore, housekeeping genes have been shown to vary considerably across different biological conditions [39]; global normalization approaches have greater stability because they exploit the abundance measures of hundreds and thousands of entities. For miRNA-seq data, this is made possible because of the large repertoire of miRNAs profiled and detected. This is in contrast to qPCR profiling, where often, only a limited number of targets are assayed. As a result, housekeeping miRNAs must be used for data normalization. We suspect that in experiments involving small repertoires of detectable miRNAs, such as circulating miRNAs, this may also be the case [40]. From this work, we recommend (1) the alignment of small RNA-seq data using BWA and (2) UQ or TMM for miRNA count normalization for comprehensive miRNA profiling studies.

The mapping of small RNA-seq reads to a reference genome and the subsequent annotation of miRNAs is the first step in constructing miRNA abundance profiles from NGS data. Compared with RNA-seq, spliced transcripts and indels are not relevant to miRNA alignments; however, the presence of isomiRs [41, 42] and sequencing errors need to be considered. As such, miRNA alignment cannot be performed by considering exact sequence matches only. The BWA outputs using different parameter settings are highly similar to each other and to the Novoalign results; however, the Bowtie output revealed a number of miRNAs that had higher counts in comparison, despite setting alignment parameters to be similar to BWA (see Figure 1B). The implementation of these two algorithms affects the handling of reads that do not align perfectly. For example, Bowtie did not return potential alignments where the reference sequence is shorter than the query sequence, whereas BWA returned all these possible alignments with equal scores. Because we only considered uniquely aligned reads, this resulted in lower counts for these particular miRNAs in the BWA output. Alignments performed using Bowtie 2 resulted in an even greater number of miRNAs with higher counts; examination of the alignment output revealed that many of these were attributed to the allowance of insertions and deletions. Bowtie 2, which was developed for gapped alignment, was included in our comparison to illustrate the consequences of selecting an inappropriate aligner. Gapped alignment can increase the mapping sensitivity [43], but was not appropriate for the task at hand. We focused our comparison on general-purpose aligners; however, many more tools are available for sequence alignment, which were designed and optimized for specific purposes, such as SOAPv2 for detecting and genotyping of single nucleotide polymorphisms [25, 44]. Not all aligners are appropriate for the task at hand, and we caution users of their specific choice. We find BWA may be better suited for identifying all possible alignments when a perfect match does not exist for a given query sequence.

Contrary to initial beliefs that miRNA-seq data will not require sophisticated normalization [45], we and others have observed that simply adjusting miRNA counts to the sequencing depth is inadequate [17, 18]. Even when profiling replicates, the distinct number of miRNAs identified in replicate samples may differ because of the random sampling nature of the technology; normalizing to the library size ignores this. Further scaling the data to a common reference sample (herein referred to as total count scaling) introduces more variability by pushing all samples toward the same distribution, especially for samples with different sequencing depth. While downsampling a data set to a common library size across all samples can remove some of the variance introduced by the different read depths, the increased accuracy provided by the deeper sequencing will be sacrificed (Supplementary Table S2). In accordance to the results observed by Dillies *et al*., we support the use of TMM (and UQ) for the normalization of miRNA count data. Garmire and Subramaniam, on the other hand, advocated for the use of quantile and Lowess normalization, while discouraging the use of TMM. Because TMM was not applied properly in their original publication, the authors reanalyzed their data with the correct implementation [46, 47]. Despite an improvement in performance, the overall conclusions remained the same; their results could be attributed to the combination of their choice of data sets and evaluation metrics. Our overall comparison of the different combinations of alignment output and normalization procedures suggested that UQ, TMM, DESeq, cyclic loess and quantile normalization are highly similar (Figure 5B). However, quantile and cyclic loess normalization may be too aggressive by forcing the distribution of the samples to be the same (Figure 2A), regardless of the presence of samples that may have inherently different distributions. In addition, increased variability was noted in the lower abundance miRNAs compared with UQ and TMM normalized data.

To ensure comparability across different miRNA-seq studies, a standardized data preprocessing pipeline should be established. To this end, we have conducted the present study to evaluate small RNA-seq data preprocessing procedures with the end-goal of retrieving miRNA abundance profiles. The combinations of different aligners and normalization methods were assessed in terms of variance, bias and accuracy for the differential expression analysis of miRNAs based on fold-change estimates. Based on these evaluation criteria, we conclude that the alignment of sequencing reads and the subsequent normalization of the miRNA count data using BWA with one mismatch across the entire read and UQ or TMM, respectively, lead to more accurate results in downstream analyses. We also note that the output from different aligners is not necessarily the same—a topic which has not been previously explored for small RNA-seq data. Finally, we make available data with spike-in controls that can be used for the future development of methods for the preprocessing and analysis of miRNA-seq data.

## Supplementary data

Supplementary data are available online at http://bib.oxfordjournals.org/.

**Key Points**

- In recent years, a number of tools have been made available for the preprocessing of small RNA-seq data to retrieve miRNA abundance profiles. Different methods will affect the outcome of downstream analyses. Despite this, no standard pipeline has been implemented.
- Previous comparative studies evaluating different normalization procedures resulted in conflicting conclusions.
- A spike-in dilution study was designed to evaluate the effects of different aligners and normalization methods with respect to the final miRNA count data distribution, variance, bias and accuracy of differential expression analysis.
- We recommend the use of BWA for alignment of small RNA-seq data to recover miRNA abundance profiles, followed by TMM or upper quartile scaling for normalization before conducting any downstream analyses.

Supplementary Data
