# Supplementary material for: Optimization of miRNA-seq data preprocessing
Source: Brief Bioinform. 2015 Apr 17;16(6):950–63. doi: 10.1093/bib/bbv019 (PMC4652620; doi:10.1093/bib/bbv019)
Supplement: Supplementary Data [file supp_16_6_950__index.html]

Optimization of miRNA-seq data preprocessing — Optimization of miRNA-seq data preprocessing — Supplementary Data 

# Optimization of miRNA-seq data preprocessing

## Supplementary Data

files

**Files in this Data Supplement:**

- Supplementary Data - pdf file
