# Supplementary material for: Optimization of miRNA-seq data preprocessing
Source: Brief Bioinform. 2015 Apr 17;16(6):950–63. doi: 10.1093/bib/bbv019 (PMC4652620; doi:10.1093/bib/bbv019)
Supplement: Supplementary Data [file supp_bbv019_Supplementary_Material.pdf]

Supplementary Material

Supplementary Tables 2
Table S1. Spike-in Sequences 2
Table S2. Sensitivity, Specificity and Accuracy of Differential Expression Analysis...... 3
Supplementary Figures 4
Figure S1. Distribution of Reads Annotated Across the References/Databases. 4
Figure S2. Comparison of Sequencing Depth. 6
Figure S3. Density Distribution of Raw and Normalized Data. 9
Figure S4. Variance Comparison. 10
Figure S5. Bias Assessment: Comparison to qPCR. 11

Supplementary Tables

Table S1. Spike-in Sequences
The following sequences were spiked into a common background reference for the assessment of bias.

Name	Sequence
ath-miR159a	UUUGGAUUGAAGGGAGCUCUA
ath-miR166a	UCGGACCAGGCUUCAUUCCCC
ath-miR169h	UAGCCAAGGAUGACUUGCCUG
ath-miR173	UUCGCUUGCAGAGAGAAAUCAC
ath-miR401	CGAAACUGGUGUCGACCGACA
ath-miR403	UUAGAUUCACGCACAAACUCG
ath-miR405a	AUGAGUUGGGUCUAACCCAUAACU
ath-miR771	UGAGCCUCUGUGGUAGCCCUCA
ath-miR835-5p	UUCUUGCAUAUGUUCUUUAUC
ath-miR1888	UAAGUUAAGAUUUGUGAAGAA
ath-miR3434*	UCAGAGUAUCAGCCAUGUGA

Table S2. Sensitivity, Specificity and Accuracy of Differential Expression Analysis. The sensitivity, specificity and accuracy of differential expression analysis relative to qPCR was computed for data processed by the different combination of aligners and normalization methods.

a. accuracy

	novoalign	bwa_seed	bwa	bowtie_seed	bowtie	bowtie2	subsampled
**raw**	0.79	0.80	0.80	0.79	0.80	0.84	0.82
**cpm**	0.82	0.84	0.84	0.84	0.84	0.84	0.82
**total count scaling**	0.80	0.82	0.82	0.82	0.82	0.84	0.82
**UQ**	0.79	0.84	0.82	0.82	0.82	0.82	0.79
**TMM**	0.79	0.82	0.84	0.82	0.84	0.82	0.79
**DESeq**	0.79	0.80	0.80	0.80	0.80	0.84	0.79
**linear regression**	0.79	0.79	0.77	0.77	0.77	0.84	0.80
**cyclic loess**	0.82	0.84	0.84	0.84	0.84	0.88	0.82
**quantile**	0.82	0.84	0.84	0.84	0.82	0.84	0.82

b. specificity

	novoalign	bwa_seed	bwa	bowtie_seed	bowtie	bowtie2	subsampled
**raw**	0.86	0.86	0.86	0.81	0.86	0.76	0.81
**cpm**	0.86	0.86	0.86	0.86	0.86	0.76	0.81
**total count scaling**	0.81	0.81	0.81	0.81	0.81	0.76	0.81
**UQ**	0.86	0.86	0.86	0.86	0.86	0.76	0.81
**TMM**	0.86	0.86	0.86	0.86	0.86	0.76	0.81
**DESeq**	0.81	0.81	0.81	0.81	0.81	0.76	0.81
**linear regression**	0.86	0.86	0.86	0.86	0.86	0.76	0.81
**cyclic loess**	0.86	0.86	0.86	0.86	0.86	0.81	0.86
**quantile**	0.81	0.81	0.81	0.81	0.81	0.76	0.76

c. sensitivity

	novoalign	bwa_seed	bwa	bowtie_seed	bowtie	bowtie2	subsampled
**raw**	0.74	0.77	0.77	0.77	0.77	0.89	0.83
**cpm**	0.80	0.83	0.83	0.83	0.83	0.89	0.83
**total count scaling**	0.80	0.83	0.83	0.83	0.83	0.89	0.83
**UQ**	0.74	0.83	0.8	0.80	0.80	0.86	0.77
**TMM**	0.74	0.80	0.83	0.80	0.83	0.86	0.77
**DESeq**	0.77	0.80	0.80	0.80	0.80	0.89	0.77
**linear regression**	0.74	0.74	0.71	0.71	0.71	0.89	0.80
**cyclic loess**	0.80	0.83	0.83	0.83	0.83	0.91	0.80
**quantile**	0.83	0.86	0.86	0.86	0.83	0.89	0.86

Supplementary Figures

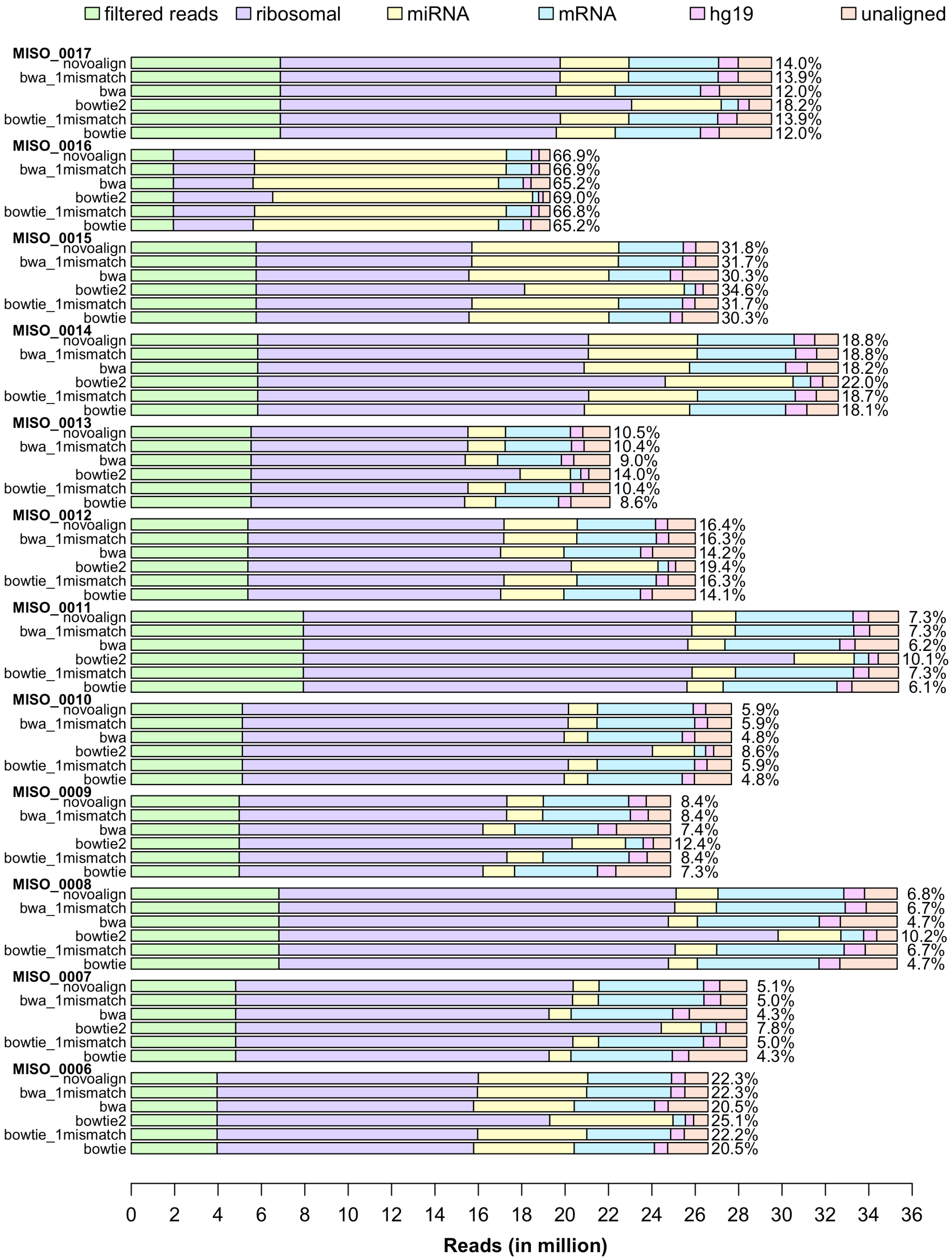

Figure S1. Distribution of Reads Annotated Across the References/Databases. Following the sequential alignment of sequence reads to different references, including ribosomal RNA, miRNA, refSeq, and the human genome (hg19 random), the number of

reads annotated to each reference by the different aligners is compared. The percentage value indicates the percentage of miRNAs recovered. All alignment algorithms recovered similar proportion of reads to each respective reference, except for Bowtie 2, which aligned a much larger number of reads to each database.

a)

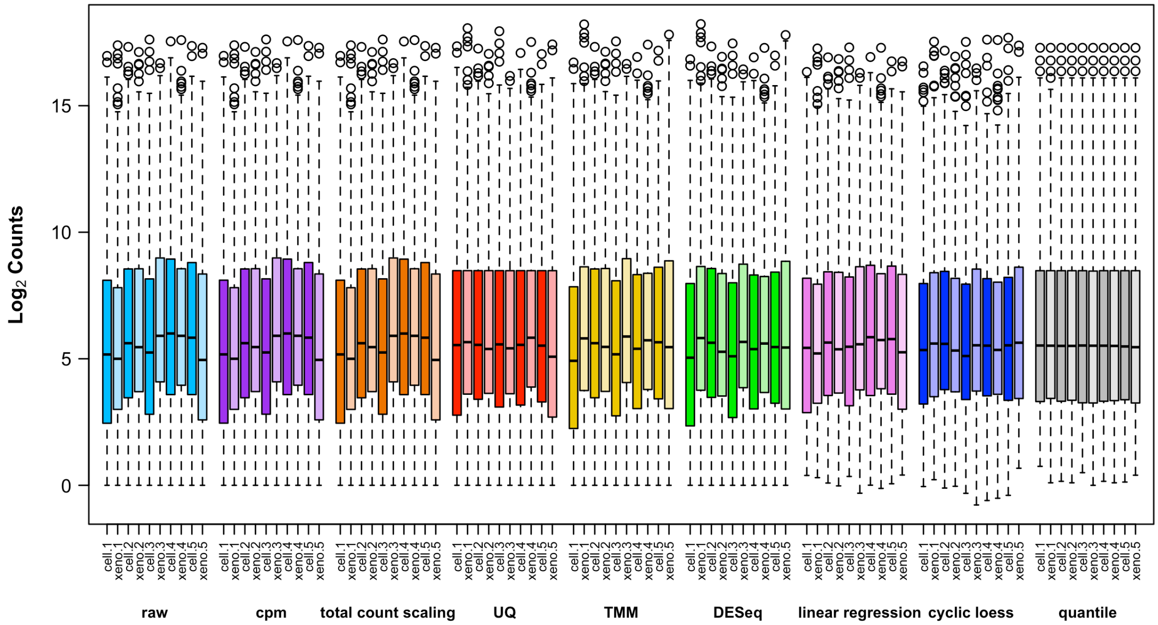

b)

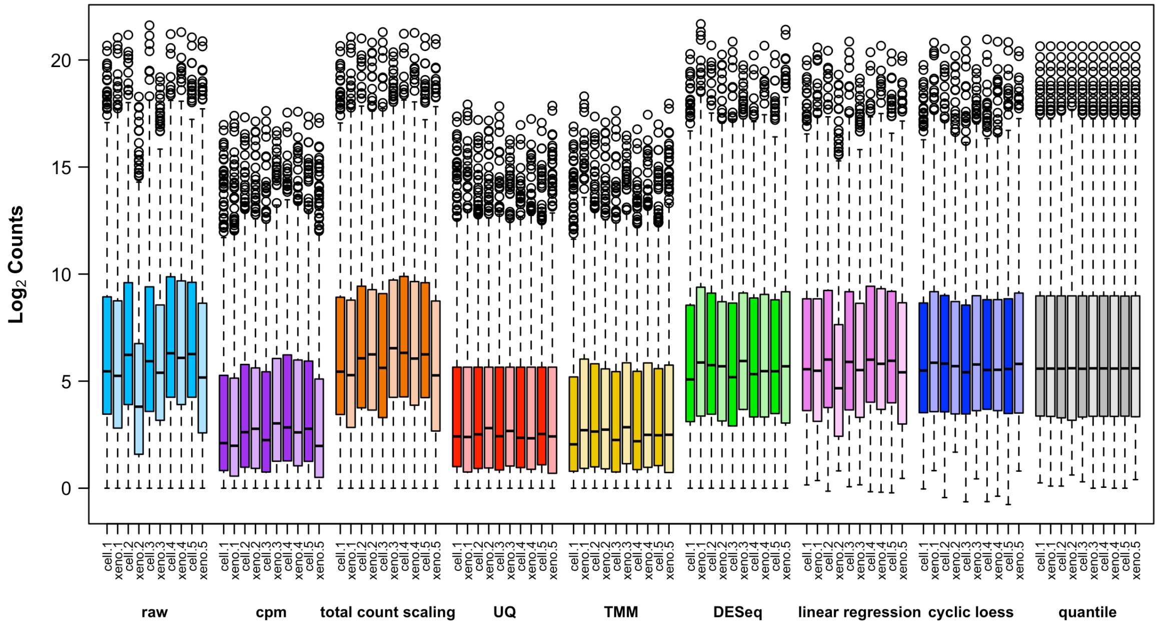

Figure S2. Comparison of Sequencing Depth.

a) To simulate a dataset with similar read depth across all samples, subsampling was performed on each sample from the cell lines-xenografts comparison study to recover 1M reads per samples. The boxplots show the distribution of reads of the raw and normalized data. When library sizes are equal across a dataset, the raw and normalized data, regardless of the normalization technique used, show stable count distributions. Normalization

methods are represented by different colors, while the two biological classes are distinguished by the dark and light shades for cell lines and xenografts, respectively.
b) When large differences in library size exist, not all normalization method produce comparable read distributions. For example, sample xeno.2 has a much lower read depth compared to all other samples. This effect is not removed by linear regression normalization.

a)

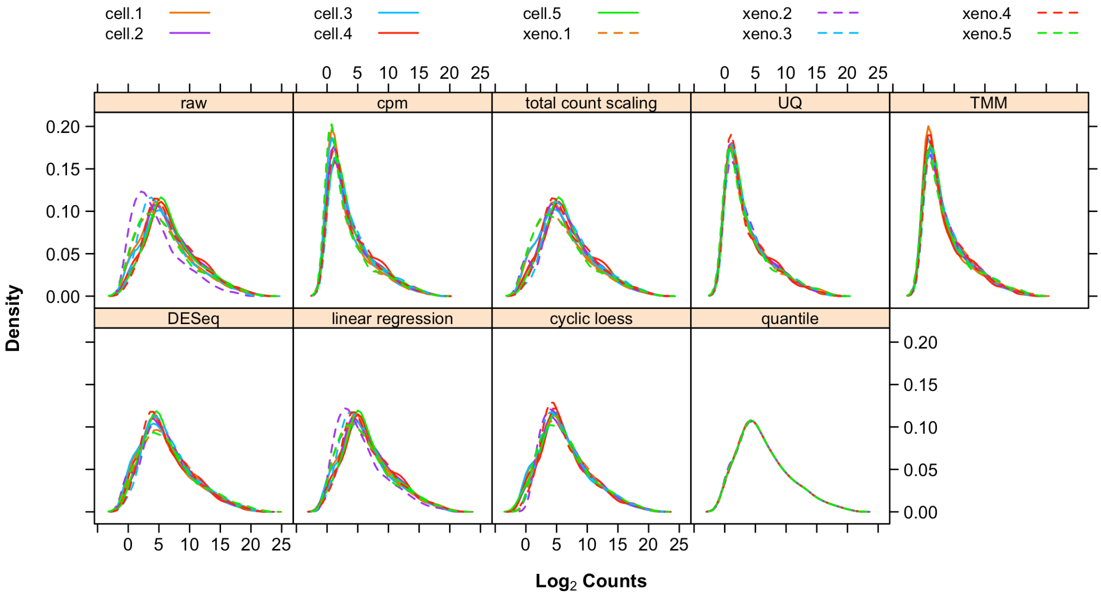

cell.1
cell.2
cell.3
cell.4
cell.5
xeno.1
xeno.2
xeno.3
xeno.4
xeno.5
raw
cpm
total count scaling
UQ
TMM
DESeq
linear regression
cyclic loess
quantile
Density
Log2 Counts
0.20
0.15
0.10
0.05
0.00
0
5
10
15
20
25

b)

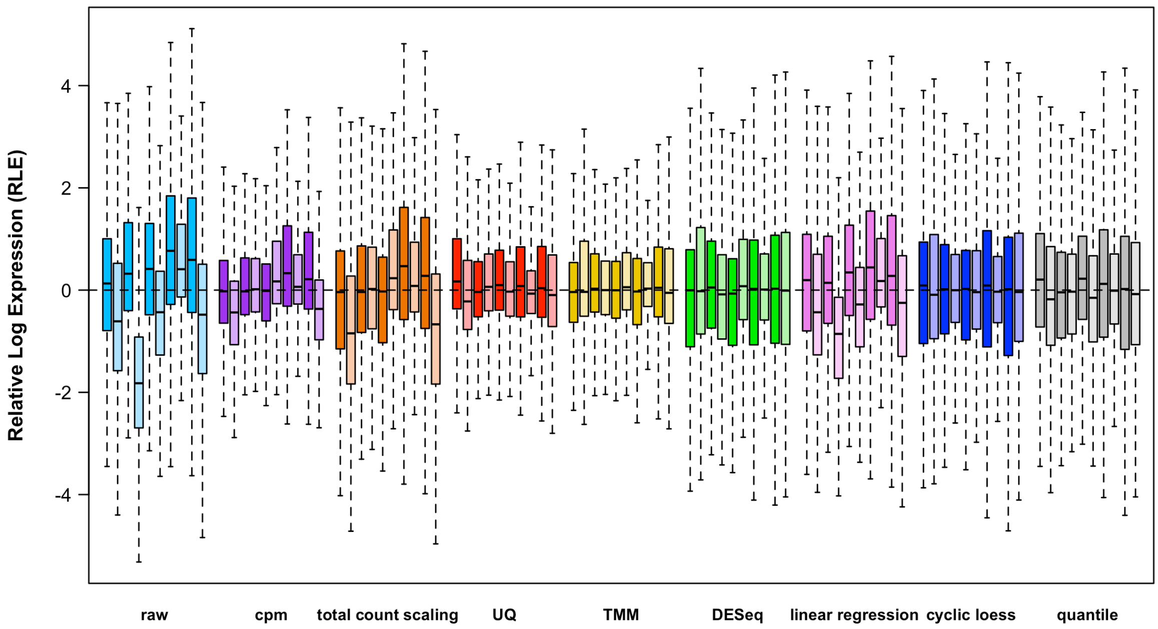

Relative Log Expression (RLE)
4
2
0
-2
-4
raw
cpm
total count scaling
UQ
TMM
DESeq
linear regression
cyclic loess
quantile

c)

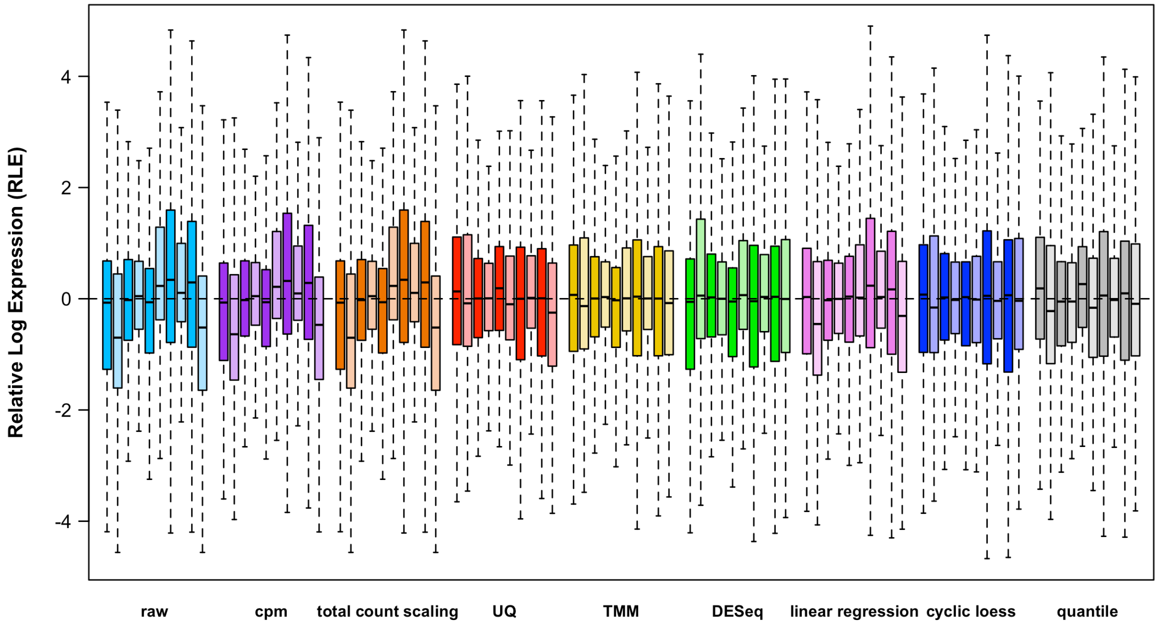

Figure S3. Density Distribution of Raw and Normalized Data.

a) Distributions of the samples before and after normalization are shown in different panels for each normalization method. Samples are shown in the same color across the plots. The density distribution curves show that UQ and TMM normalization result in similar distribution across all samples. Quantile normalization forces the distribution of all samples to be the same, whereas more variability across the samples remains when all other methods are used. Samples are represented by the same color across the panels.

b) The relative log expression (RLE) values derived from properly normalized data should be centered at zero and have comparable distributions across similar samples. Only data normalized by UQ and TMM have tighter distributions of relative log expression values centered at zero. This is in accordance to the results observed in the spike-in dataset.

c) For the subsampled data, the RLE distributions are comparable across all methods, with values centered at zero. When small differences exist in library size, the different normalization techniques perform similarly.

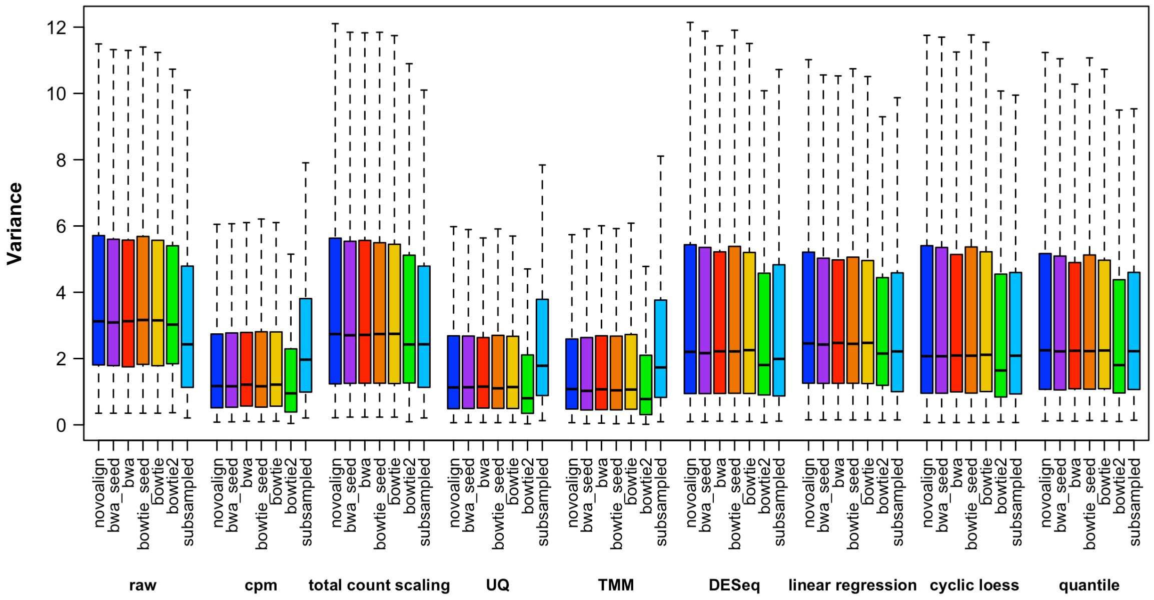

Figure S4. Variance Comparison.
In this series of boxplots, the data subsampled to a common read depth of ~2.2M reads was also included. The variance of the log2 counts of all miRNAs was computed across the samples. Although a decrease in variance is observed in the subsampled data normalized by cpm, UQ and TMM, the decrease is not as large as observed in the original dataset, which had large differences in sequencing depth.

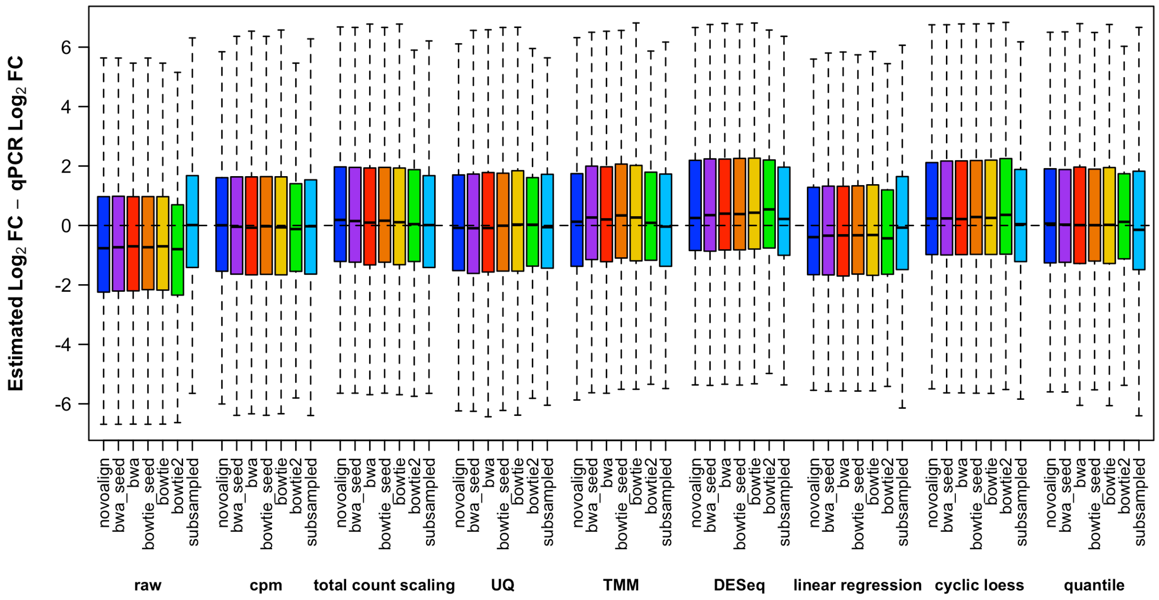

Figure S5. Bias Assessment: Comparison to qPCR.

The difference between fold-changes determined using miRNA-seq data and qPCR data was determined for 56 miRNAs. The boxplots are grouped according to the normalization methods, with colors representing different aligners and the subsampled data. The log2 ratios determined using the unnormalized raw miRNA counts overestimates the differences between cell lines and xenografts when compared to the qPCR data. Cpm, UQ and quantile normalization effectively reduces this bias. In the subsampled raw counts, while the distribution of the differences between the sequencing and qPCR log2 ratios is centered at zero, DESeq and quantile normalization increased the bias.
